# Do urban tourists prefer vegetarianism? An urban-rural comparison of vegetarian consumption in China

**DOI:** 10.3389/fnut.2022.996158

**Published:** 2022-12-08

**Authors:** Guoyi Chen, Wei Tan, Ning Ran, Jiansheng Zhang, Bangquan Yan

**Affiliations:** ^1^Department of Business Management, Chongqing Three Gorges University, Chongqing, China; ^2^Department of Public Administration, Chongqing Three Gorges University, Chongqing, China; ^3^Department of Financial Management, Chongqing Three Gorges University, Chongqing, China

**Keywords:** vegetarian, Generation Z, rural, urban, contrast

## Abstract

The adoption of a vegetarian diet might have public health and environmental benefits. However, little is known about urban and rural Generation Z tourists' attitudes toward vegetarianism or vegetarian consumption within the Chinese urban and rural settings. Hence, to address this gap, the present study adopted a sequential and mixed research approach based on a survey (*n* = 212) and laddering interviews (*n* = 20) to validate post-millennial tourists' motives for adopting a vegetarian diet. The results identified the top four motives as environmental protection and resource conservation, ethical consideration, personal taste and choice, and personal healthcare issues. The top four barriers to vegetarianism were unavailability and limited choice, peer pressure, traditional prejudice/habit, and the inability to change. The results also demonstrated that both rural and urban tourists adopt vegetarianism mainly for environmental protection and ethical consideration, a subtle difference between them is that urban vegetarians emphasized ethical considerations more but rural ones emphasized food and variety. Urban consumers considered unavailability and limited choice as the topmost barriers to being vegetarian, while rural vegetarians found traditional prejudice to be restricting. Due to traditional dietary habits and peer influence, rural tourists face many more challenges when adopting a vegetarian diet. Understanding the perceived benefits and barriers to being vegetarian in different regions will not only enrich the theory of food nutrition but also expand Generation Z tourists' consumption behavior and practices.

## Introduction

Tourism and food consumption have become a new focal point of Chinese economic growth within the country's economic reform. Since 1978, China's urban-rural duality has also come to be reflected in the consumption patterns of residents, particularly food consumption. Based on food consumption data from the National Bureau of Statistics of China, grains account for the highest proportion, followed by meat; however, the demand for meat has increased year by year. Though the consumption of meat among urban residents is relatively high, it shows a downward trend year by year (1). In the past 10 years, even as urbanization has continued, the urban-rural differences persist and the food consumption structure between them is gradually widening. Urban residents have a higher tendency to consume fruits and vegetables rather than merely meat. Several studies showed that urban residents widely exposed to the newest concepts of food nutrition, personal healthcare, and environmental protection are easily inclined to accept vegetarianism (1, 2). Thus, are there any differences in vegetarians among urban and rural populations, and do urban residents have a higher proclivity toward vegetarianism than rural ones?

This study aimed to address this issue, and the goal of this research is to investigate the differences between urban and rural vegetarianism by analyzing the motivations, benefits, and barriers to following a vegetarian diet during travel. In the present study, vegetarian refers to individuals who eat a diet consisting wholly of vegetables, fruits, grains, nuts, and sometimes eggs or dairy products, and they do not consume meat, fish, and poultry. Well-planned vegetarian diets have proved to be healthy and nutritionally adequate and they can provide enough nutrition for people at all stages of life (3, 4). There are quite a large number of benefits to following a vegetarian diet. According to a survey by Izmirli and Phillips, a meat-free diet can directly lower the intake of calories as compared with non-vegans, and this can assist people who want to lose weight (5). Second, vegetarians tend to have fewer intakes of fat, saturated fat, and cholesterol (6). Finally, vegetarian diets contain rich nutrition, such as vitamins, folate, photochemical, potassium, fiber, and protective compounds, all of which are beneficial for human beings (7, 8).

Although there are benefits to consuming vegetarian products, the proportion of the vegetarian population in China is still small; among 1.3 billion people at present, only 3.8% of the population consider themselves vegetarian (6).

Generation Z, born between 1995 and 2010, gradually became the main influence on the food consumption market, and they play an important role in vegetarian consumption in China (9). Generation Z advocates freedom and is obsessed with tourism. They are loyal fans of rural tours, RV tours, camping, outdoor tours, and music tours. However, because vegetarianism is not yet widespread in China, choices are very limited, and finding enough comfortable vegetarian food becomes a primary concern during the journey. In addition, because of China's typical urban-rural duality (10), the vegetarian consumption gap between them is still widening. Thus, the purpose of the present study is to contrast the perception of vegetarian diets between urban and rural Generation Z tourists and explore their attitudes, motivations, driving factors, and barriers to following a vegetarian diet. In this context, the key research questions are:

(a) What are the most commonly perceived benefits of vegetarianism for urban and rural Chinese Generation Z tourists?
(b) What are the most commonly perceived barriers to vegetarianism for urban and rural Chinese Generation Z tourists?
(c) What are the main differences in terms of perceived benefits and barriers between them and why?

This study is important because it attempts to identify the new generation's attitude toward a vegetarian diet, by investigating their perception of the benefits and barriers to adopting a vegetarian diet. From the perspective of nutrition and dietetics, the information from this study could be beneficial for enhancing nutritional interventions and contributing to the reduction of certain chronic lifelong diseases in Chinese societies (11).

## Literature review

This study aimed to undertake a contrast analysis of post-millennial tourists' attitudes toward vegetarianism, including perceived benefits and barriers. This section provides a brief review of perceived benefits and barriers.

### Motivations and perceived benefits of vegetarian consumption

As people become more and more concerned about health, environmental protection, and animal welfare, vegetarianism has increased and has drawn increasing attention from theory and practice, in particular psychology research, though it is still at an early stage. Psychological processes regarding vegetarianism involve cognitive, emotional, and motivational aspects and vegetarian identity. This study aimed to investigate the factors influencing post-millennials motivation for adopting vegetarianism.

Studying their motivation provides a new way of understanding the driving forces behind their vegetarian choice (12). Table 1 presents a general summary of nine representative studies examining the consumers' motivations for adopting vegetarian diets. Among them, two studies chose a qualitative approach and had open-ended questions to identify the motivations, and the remaining seven studies adopted quantitative research methodology using a closed-ended questionnaire for examining respondents' motivations.

**Table 1:** List of previous researches on the benefits and motivations of vegetarian diet.

**References**	**Research sample**	**Research method**	**Results: Main motive(s)**
Waldmann et al. (13)	154 vegans Germany	Quantitative Questionnaire: Closed-format question Research was carried out through Journal advertising	Among all the 154 respondents, 48.7% (75 persons) were driven by health-related reasons, 41.6% (64 persons) were motivated by ethical-moral issue, these two motives occupied majority, the other motives were selected relatively limited. Among them, only 2 persons chose hygiene motivator and only 1 person chose Environment-related reasons (13)
Fox and Ward (14)	33 participants from US, Canada and the UK.	Qualittive Questionnaire: open-format question It conducted online survey of open-ended questions, and then a follow-up e-mail interviews with 18 participants	Result indicated that health and ethical issue were the top driving factors for participants' vegetarianism, and only one case was environmental-related motivator (14).
Dyett et al. (15)	100 vegans United States of America	Quantitative Close-format question Data collected through Printed advertisement	Results shows that 47% of the participants chose vegan diet for health-related motivation, 40% of them for moral motivation, 9% of them for religious beliefs, 2% chose it for environmental motivation, and the last 2% chose it for family or other type of reason (15).
Rothgerber (16)	315 vegans and 200 vegetarians	Quantitative Questionnaire Close-format question Collect on internet	Among 315 vegans, 177 persons (56.2%) were driven by ethical motives, 40 persons (12.7%) were driven by personal health-related issue, and the last 98 chose (31.1%) vegan diet for other motives (16).
Radnitz et al. (17)	246 vegans United States of America, Canada and other countries	Quantitative Questionnaire: Close-format question Data collected by Vegan events and social media	Researchers just explored two kinds of motivations: Ethical reasons and health-related reasons. And result showed that 81.7% (201 persons) chose ethical motivators and 19.3% (45 persons) chose health-related motives (17).
Kerschke-Risch (18)	852 vegans Germany	Quantitative Questionnaire: Close-format question Data collected through Internet, and the sample method was snowball sampling	Participants were asked to rank different factors related to their influence on their decisions for quitting meat, Likert list was adopted for “1 means no influence at all” to “5 means very strong influence”. The result showed that the index score of climate protection was 3.8, the index score of health-related reason was 3.2 and the index score about factory farming (ethical consideration) was 4.4 (18).
Janssen et al. (19)	329 vegans Germany	Quantitative Open-ended question	Among all the 329 vegans, 89.7% were driven by animal-related reasons, 69.3% were motivated by personal health related motivations and 46.8% were driven by environmental related motivations (19).
Dorard and Mathieu (20)	49 vegetarians and 52 omnivores France	Quantitative research close-ended questionnaires Data collected by Facebook	Results indicated that compared with the omnivores, the motivations of vegetarians were more related with health (*p* = 0.001) and natural content (*p* < 0.0001), less related with weight control motivations (*p* = 0.015) (20).
North et al. (21)	701 participants 371 vegans, 99 Vegetarian and 231 Omnivore Australia	Qualitative Open-ended question Data collected through Online survey of Qualtrics	The participants were divided into three groups, and similar motivation among them is health-related reason. The second one is environmental protection, and the animal welfare was also cited most by vegans and vegetarians. Taste and enjoyment for diet were also identifies as motivation (21).

Based on previous research, the most frequently mentioned motivations for adopting a vegetarian diet included ethical considerations, environmental protection, health, individual taste, and religious reasons (13–15). The ethical reason, also referred to as moral reason, indicates that people should consider the following when determining their dietary preferences: animal welfare, animal right, and their suffering during livestock production (13). Environmental concern includes the ecological reason for choosing a vegetarian diet, advocating environmental protection, resource-saving, and the greenhouse effect (14), which were also classified under ethical reason in the past. Health-related reasons uphold that a vegetarian diet is beneficial for personal health while compared with omnibus or purely meat consumption (15), arguing that it can prevent people from common illnesses and is good for personal fitness (16, 17). Of the three motives, ethical and environmental motives were considered to be of public interest, and the health-related motive was assumed to be guided to a greater extent by self-interest (18).

However, some researchers also pointed out that the classification of motives to adopt vegetarianism into just two groups was not feasible, for there might be other kinds of motives that might have been overlooked, such as personal taste and food choice, personal interests, and family lifestyle (19). These multiple motives may complement and reinforce each other for stimulating people to adopt a vegetarian diet. Based on the above, motivations can be classified as those driven by personal health, personal taste, environmental protection, personal interest, and ethical consideration (20, 21). Thus, we propose the following hypothesis:

H_1_: Personal health, personal taste and choices, environmental protection and resource conservation, personal interests, and ethical consideration are positively related to Generation Z tourists' willingness to follow a vegetarian diet.

This classification and differentiation have been described and explained by Greenebaum, who also stated that ethical and environmental-related vegetarians are different from personal-related vegetarians (22). The latter were considered self-interest-oriented and hedonistic vegetarians, while the former was marked as public-interest-oriented and altruistic vegetarians, and they may have quite a different understanding and feeling about adopting a vegetarian diet. In fact, in most cases, ethical and environmental protection-related motivations occupied a majority of the vegetarian community. Especially in China, which is considered an oriental traditional country favoring collectivism, people are encouraged to put public interest, including environmental protection and animal welfare, as their first priority (23). Under these circumstances, we propose the following hypothesis.

H_2_: Of all the benefits, ethics-related and environmental protection-related motives have a stronger positive effect on willingness to be vegetarian.

Although vegetarianism is not mainstream in China, there is a growing trend toward reducing meat consumption. Especially for urban Generation Z, who have been living in cities or towns for a long time, being exposed to the concepts of environmental protection, animal protection, and personal healthcare, and fully understanding the benefits of vegetarianism and vegetarian consumption, they were more motivated by the perceived benefits of vegetarian consumption (24). In addition, urban areas have a long history of vegetarianism compared to rural areas. Thus, based on the above, we propose the following hypothesis.

H3: The positive influence of ethics-related and environmental protection-related motives is stronger for tourists in urban areas than those in rural areas.

### Perceived barriers to vegetarian consumption

In 2003, Lea and Worsley surveyed the perceived benefits and barriers to the consumption of a vegetarian diet in Australia (25). In this study, over 1,000 south Australians were randomly selected and required to fill out a questionnaire consisting of 49 questions, among which 25 questions were about personal barriers and 24 items were about benefits. Of the 1,000 respondents, 70.6% of them completed the questionnaire. It was found that only 1.5% of them identified themselves as vegans and 7.2% as semi-vegans (25). And the main barrier for them to adopt a vegetarian diet was the enjoyment of eating meat and the unwillingness to give it up. The second barrier was the lack of enough information about vegetarianism. Traditional beliefs that people are meant to eat meat were the third barrier to meat consumption. Health concerns were also noted as a barrier for vegetarians who did not eat any meat. Overall, respondents' enjoyment of eating meat and their unwillingness to change their diet were considered the biggest barriers to the consumption of a vegetarian diet. Lea and Worsley (25) study concluded that there were many Australians who were quite interested in vegetarianism, and they strongly believe that vegetarian consumption was positively related to health benefits. Because the enjoyment of meat is the biggest barrier to adopting the vegetarian diet, it was suggested that the most feasible and suitable way was to provide both meat- and plant-based diet instead of completely eliminating meat input (25).

In 2006, Lea et al. conducted a study exploring people's attitudes toward the consumption of a plant-based diet. More than 1,000 adult respondents were selected randomly and 51% of them completed the questionnaire. Of these 62% demonstrated high or somewhat interest in consuming a plant-based diet (26). The main perceived barrier to adopting a plant-based diet was the lack of enough information and consumers had relatively few choices. Other common barriers included people's unwillingness or inability to change their family's diet, and they were also reported to have relatively low availability of plant-based options while eating out. This research also showed that as a community, they may be unfamiliar with the notion of a plant-based diet, which was an unexpected prevalent barrier to plant-based consumption.

Wieliczki also examined the main perceived barriers to vegetarianism in universities (27). The research explored how university students had enough knowledge about vegetarianism and also identified the biggest differences in the field of perceived barriers between vegetarians and omnivores. The respondents were 96 students selected using a convenience sample. The most frequently mentioned barriers to a vegetarian diet were: (a) All my family members eat meat, (b) I enjoy eating meat, (c) My friends eat meat, (d) I need more information about vegetarian food and diet, (e) I am unwilling to change my taste and habit, (f) I think that humans should eat meat, that is nature, (g) There is a relatively limited choice of a vegetarian diet while eating out, (h) I do not have enough right to change my eating habit, (i) My family members/partners/friends/relatives do not want to eat any vegetarian food, and (j) It is uncomfortable (27). A general summary of the representative research about the perceived barriers to following a vegetarian diet is presented in Table 2. Based on the above, we propose the following hypothesis:

H_4_: Traditional prejudice and habit, peer pressure, unavailability, limited choice, and unwillingness or inability to change are negatively related to Generation Z tourists' vegetarian consumption.

**Table 2:** List of previous research on the barriers to vegetarian consumption.

**References**	**Research sample**	**Research method**	**Results: Main barrier(s)**
Lea and Worsley (25)	1,000 south Australians Australians	Quantitative Closed-format question	Main barriers included a) Enjoy meat eating b) unwilling to change their current diet or routine c) traditional concept that people are ‘meant' to eat meat d) limited choices (25).
Lea et al. (26)	USA	Quantitative Closed-format question	Main barriers included a) lack of information about plant-based diets, b) unable or inability to change current food diet and eating habit C) few plant-based options (26).
Wieliczki (27)	96 subjects United States of America	Quantitative Closed-format question	Ten most common perceived barriers identified, in order, are: a) my family members prefer meat eating b) I like meat eating) my friends eat meat d) I am in need of more information about vegetarian diets e) I am unwilling to change my eating habits f) I think humans are meant to eat meat g) there's limited vegan diet choices when I eat out h)I don't have enough will power, i) my family/spouse/partner won't eat vegetarian foods, and j) it is inconvenient (27).
Radnitz et al. (17)	246 vegans USA, Canada and other countries (not further specified by the authors)	Quantitative Closed-format question Data collected by Vegan events and social media	•Unable to change the current diet •Lack of enough choice of substitute •Inconvenient (17).
Mullee et al. (28)	2436 participants Belgium	Quantitative Online questionnaire with multiple-choice questions	Key barriers for not being vegetarian included inadequately tasty, lack of interest and awareness and limited choices (28).
Rosenfeld and Tomiyama (29)	579 participants United States	Quantitative Closed-format question	Key barriers including inadequately tasty, inadequately nutritious, inconvenient consumption, high price and socially stigmatizing (29).
Beningfield et al. (30)	458 participants South Africa	Quantitative Closed-format question Cross-sectional study	Most frequently perceived barriers identified, they are: a) I like eating meat b) Except meat, I don't know eat what c) Somebody else decides what I eat everyday d) My Family members eat meat e) Eating meat is favorable in my culture f) I think humans are meant to eat meat h) I don't want people to think I am strange i)Limited vegetarian choice when I eat out (30).

In 2017, Radnitz et al. conducted quantitive research in Belgium where 2,436 participants were invited to complete an online questionnaire with multiple-choice questions. The results indicated that the key barriers to not being vegetarian included not being tasty enough, lack of interest and awareness, as well as limited cooking choices (17, 28). Similarly, in 2020, Rosenfeld and Tomiyama found that not being tasty enough, inadequately nutritious, inconvenient consumption, high price, and socially stigmatizing were the main barriers to adopting a vegetarian diet (29). A majority of the participants reported that limited plant-based options as the main barrier. More than half of the respondents admitted that the obvious benefits included improvement in personal health by adopting a vegetarian diet, while on the other hand, most family and friend members could not resist the temptation of a meat diet, which may inhibit the adoption of a vegetarian diet (30). Furthermore, as can be seen from Table 2, the principal barrier for vegetarians was unavailability and limited choice, followed by peer pressures, and traditional habits. In other words, limited options together with peer pressure inhibit consumers to adopt vegetarian diets.

Based on the above, we propose the following hypothesis:

H_5_: Among all the barriers, unavailability and limited choice, peer pressure as well as traditional habits and prejudice have a stronger negative influence on vegetarian diet consumption.

Previous scholars argued that manual laborers rely more on meat for providing strength and stamina (31). Most rural populations are mainly engaged in manual labor and, therefore, are more inclined to consume meat. Meat consumption forms a significant portion of rural citizens' daily consumption (32). Second, the existence of a dual economy structure (between the urban and rural) results in the more obvious income and consumption differences, which leads to poor infrastructure and limited vegetarian choices in rural areas (10). Third, according to Hofstede's cultural dimensions, China as an oriental traditional country favoring collectivism (33), and rural citizens may experience more pressure to continue with traditional habits. Based on the above, we propose the following hypothesis:

H_6_: The negative influence of traditional prejudice and habit and unavailability and limited choice are higher for tourists in rural areas than those in urban ones.

As a new generation living in an information era, urban Generation Z has convenient access to recent diets and healthcare information through mobile internet, and widespread information helps them to have a deeper understanding and are, therefore, prone to accept vegetarianism (34). In addition, due to the profound influence of foreign vegetarian culture, the urban area has a longer history of vegetarian consumption than rural areas (35). Therefore, urban youth are prone to accept vegetarianism easily. While on the other hand, previous scholars have argued that rural populations, mainly consisting of manual laborers and other working-class members, are especially prone to perceive the consumption of meat as a key component of maintaining strength (31, 36). Thus, we propose the following hypothesis:

H_7_: Urban tourists (vs. rural ones) have a higher proclivity toward vegetarian consumption.

## Materials and methods

To identify Generation Z tourists' perception toward a vegetarian diet, especially their attitude, motivation, driving factors, and barriers to accepting a vegetarian diet, the research adopted mixed research methods (including both qualitative and quantitively methods) where the quantitative findings from the questionnaire were cross-checked against the qualitative findings from the focus groups enhancing the validity of the research (37).

### Questionnaire design and survey implementation

A questionnaire was adapted from previous studies by Lea and Worsley, Clarys et al., and Hawkins et al. (25, 38, 39). The questionnaire was organized into three parts. The first part addressed 24 perceived benefits of following a vegetarian diet and the second part had 26 perceived barriers. A five-point Likert scale ranging from 1 to 5 (1 = “strongly disagree” and 5 = “strongly agree”; Cronbach's α = 0.89) was used. The third part contained four closed questions and one open-ended question about demographics, such as native place, sex, age, and education major, as well as questions about participants' current diets. Multiple-choice questions were also included in this part to figure out the most important perceived motivations and barriers.

Most of the questions were designed to measure the variables within the hypotheses. These questions were presented as statements and participants were asked to express their attitude toward these statements by using a Likert scale. The 5-point Likert scale is considered suitable for this investigation as it allows the researcher to measure participants' opinions and attitudes toward the statements made (40).

A pilot study was administered for increasing the feasibility and readability of the survey. The pilot questionnaire was compiled with broad open-ended discussion prompts (40). The author, however, did not follow this framework or guidelines rigidly. The discussion was conducted freely so that ideas could emerge and be adequately probed. The results of the pilot study were used to make improvements to the original questions.

The target population for this study was a convenience sample composed of 212 undergraduate and graduate students from Chongqing Three Gorges University who claimed to be vegetarians. The underlying reason for selecting tourism-related students was that most of them had made more than four trips in the past, and they had an intensive perception of motivations and barriers to vegetarianism during their travels. In addition, as a part of the university's Professional Talent Cultivation Plan, all tourism-related students are required to participate in a tour guide internship program conducted each year at different tourist attractions across the country. As instructors of this program, the authors had ample chances to contact these Generation Z and request their participation in the study and collect primary data during their internship.

As to the data collection procedure, the researchers first delivered the survey information on social media like Weibo and WeChat, the requirement, instructions, and procedures of this survey were also sent out simultaneously inviting Generation Z who claimed to be vegetarian. Secondly, the researchers also contacted the class monitor of each internship class and asked them to forward the survey link to their classmates and contemporaries. After identifying qualified participants, the class monitors provided the researchers with a list of potential participants. Thirdly the researchers contacted the potential participants and invited those qualifying among them to participate in the survey. Respondents from different provinces were invited for ensuring representativeness and universality. Fourthly, after obtaining oral approval from the respondents, the researchers briefed them about the questionnaire survey. Lastly, the respondents completed the questionnaire, and the primary data was collected on the spot. A total of 212 respondents took part in the questionnaire survey and among them, 90 were from rural regions and 122 were from urban regions. Urban respondents were those who were permanent residents or lived in urban areas in the past, and rural respondents were those who were living in rural areas. The whole survey was completed in May 2022.

### Data analysis

All the collected data were arranged in a form, in which each question was marked with a certain number, and then they were classified into different categories.

SPSS 2016 was used to interpret the quantitative data because its functions are sufficient for the analysis of this study. Tables with mean value analysis and analysis of variance (ANOVA) are useful and effective tools to identify the important benefits and barriers to adopting a vegetarian diet. To test hypotheses 1 to 4, this research conducted mean value comparison and standard deviation to describe participants' perceived benefits and barriers. This research adopted the arithmetic “mean” to calculate the average in the five groups of perceived benefits and four groups of perceived barriers.

For testing hypothesis 5, ANOVA was conducted for comparing the results between rural and urban respondents. Through ANOVA and content analysis between rural and urban respondents, the researchers were able to determine key findings that informed the conclusion.

Therefore, all the questions were analyzed through SPSS mean analysis. In addition, transcribing was necessary because it allowed the researchers to analyze participants' answers and identify the main themes that emerged during discussions.

## Results

### Sample description

The survey respondents included 90 rural participants and 122 urban participants, aged between 18 to 27, who claimed that they have always been vegetarians. This sample is consistent with the Chinese urban-rural dual structure where living standard in cities is higher than that in rural areas. There were 212 participants in total, and among them, two questionnaires were completed by flexitarians who did not meet the requirement of the study and were therefore excluded from the data analysis. Another two questionnaires were completed by vegetarians who were aged more than 27 and did not meet the age requirements of the study and thus excluded from the study. Two other questionnaires had more than 30% of the questions left blank, and were discarded. Finally, 206 questionnaires were qualified and collected for further analysis. Table 3 below summarizes the respondents' demographic profile in detail.

**Table 3:** Demographic profile.

**Demographic factors**	**Types/Ranges**	**Number of respondents**	**Percentage of respondents**
Locality	Rural	90	
	Urban	116	
Gender	Female	169	82.00%
	Male	37	18.00%
Age	18	76	36.90%
	19	38	18.40%
	20	47	22.80%
	21+	45	21.80%
Major	Tourism management	61	29.60%
	Hotel management	32	15.50%
	Business management	62	30.10%
	Tour guide	31	15.00%
	Others	20	9.70%

### Results and analysis

#### Quantitative data comparison

Table 4 presents the comparison of perceived benefits between rural and urban vegetarians. Both sides agreed that personal healthcare, personal taste and choice, environmental protection and resource conservation, and ethical consideration positively motivated participants' vegetarian consumption. However, the score of personal interest was >3.0, signifying that its effect is relatively weak. Furthermore, data indicated that urban tourists gave higher scores to the benefits of adopting a vegetarian diet, and they thought that it was good for their personal health, and vegetarian consumption could prevent diseases, improve their digestion, and provide them with plenty of food choices, and most importantly, being vegetarian was quite beneficial for environmental protection and resource conservation, and it would promote the welfare of animals. The scores of urban Generation Z were somewhat higher than that of their rural counterparts and it reflects the overall higher acceptance of vegetarianism in urban areas than that of rural regions.

**Table 4:** Comparison of perceived benefits between rural and urban respondents.

	**Rural**		**Urban**	
**Personal healthcare issue**	**Mean**	**SD**	**Mean**	**SD**
Vegetarian diets/meals help prevent disease in general	2.63	1.258	3.86	1.213
It would help me stay healthy	3.30	0.295	3.88	1.013
It would help me control my weight	3.58	1.186	3.13	1.092
It would help me improve my digestion	3.55	1.053	3.56	0.938
I would be more fit	3.42	1.205	3.62	1.127
I would have a better quality of life	3.06	1.279	3.92	1.122
I would be more content with myself	3.06	1.165	4.13	1.016
**Overall**	3.23		3.73	
**Personal taste and choice**				
It would decrease my saturated fat intake	3.97	1.005	3.41	1.151
I would eat more fiber	4.43	0.079	3.82	0.949
I would eat a more “natural” diet	3.40	1.305	3.83	1.195
I would eat lots of vitamins and minerals	3.63	1.131	3.98	0.961
I would eat a greater variety of plant foods	3.42	1.234	4.43	0.901
I would have plenty of energy	3.02	1.161	3.53	1.041
My meals will be tasty	2.78	1.224	4.06	1.025
I would have a lower risk of getting food poisoning	2.90	1.201	3.41	1.162
**Overall**	3.44		3.81	
**Environmental protection and resource conservation**				
I would contribute to environmental protection	3.76	1.215	4.56	0.981
I would contribute to resource conservation	3.59		4.35	
**Overall**	3.67		4.46	
**Personal interest**				
I would save money	2.55	1.301	2.71	2.191
I would save time	2.90	1.201	1.16	0.994
I would have fewer food storage problems	2.76	1.171	2.26	1.053
I would appear more “trendy” to my friends	2.29	1.209	2.03	1.179
**Overall**	2.63		2.04	
**Ethical consideration**				
I would promote animal welfare/rights	3.85	1.232	4.68	0.866
It would increase the efficiency of food production	3.29	1.311	4.06	1.067
It would help decrease hunger in the Third World	2.93	1.336	3.41	1.332
**Overall**	3.36		4.05	

1, strongly disagree; 2, disagree; 3, neutral; 4, agree; 5, strongly agree.

On perceived motivations from urban participants, the motivation with the highest mean score was “I would contribute to animal welfare/rights” (4.68), followed by “I would contribute to the environment” (4.56), and “I would eat a greater variety of plant foods” (4.43). In contrast, the highest mean score from rural respondents was “I would eat more fiber” (4.43), followed by “It would decrease my saturated fat intake” (3.97), and “I would contribute to animal welfare/rights” (3.95). The results show that urban vegetarians put more emphasis on environmental issues and ethical considerations, while on the other hand, rural vegetarians emphasized more on taste and diet diversity.

In addition, the multiple-choice questions on tourists' motivation for following a vegetarian diet indicated that four main factors motivated Generation Z to adopt a vegetarian diet. Of the 206 respondents, 186 (90.3%) selected ethical considerations, i.e., animal welfare and food conservation. About 86.4% of the respondents selected environmental and resource-related motivations, and 58.7% of the respondents chose personal taste and choice, which encompassed aspects related to fiber/vitamins and minerals intake, and tasty cuisine. A further 53.4% mentioned health-related motivation, i.e., motives related to staying healthy, controlling weight, and improving digestion. Interestingly, only 22% of the respondents chose personal interest-related motivation. i.e., save money, save time, and solve food storage problems. Other motivations including religious beliefs or consuming trends were cited by just 3.9% of the total respondents.

Table 5 presents the comparison of perceived barriers between rural and urban vegetarian consumers. In contrast to the perceived benefits, the results for perceived barriers showed that rural respondents gave higher scores to the barriers to adopting a vegetarian diet, indicating that rural vegetarians faced a higher degree of barriers to adopting a vegetarian diet. The data from Tables 4, 5 together indicate that urbanites have a longer history of vegetarianism than rural residents. Also, urban tourists have a much deeper understanding of vegetarianism and its consequence.

**Table 5:** Comparison of perceived barriers between rural and urban respondents.

	**Rural**		**Urban**	
	**Mean**	**SD**	**Mean**	**SD**
**Traditional perceptions and habit**				
I like eating meat	3.80	1.119	2.11	1.309
It would be (is) too expensive	3.10	1.258	2.08	1.053
There is not enough iron in vegetarian diets	3.10	1.201	1.86	1.105
There is not enough protein in vegetarian diets	3.13	1.310	1.67	1.075
There is not enough B12 in vegetarian diets	3.09	1.263	2.68	1.360
I would be (or am) worried about my health	3.15	1.176	1.73	1.000
I think humans are meant to eat meat	3.06	1.341	1.45	1.007
It is inconvenient	2.74	1.313	2.01	1.208
Vegetarian diets/meals are not filling enough	2.99	1.402	1.47	0.974
Vegetarian diets/meals are boring	2.52	1.318	1.36	0.883
I wouldn't get enough energy from vegetarian foods	2.95	1.302	1.49	0.957
**Overall**	3.06		1.81	
**Peer pressure**				
My friends eat meat	4.06	1.165	2.40	1.332
My family eats meat	4.15	1.105	2.70	1.429
My family/spouse/partner won't eat vegetarian meals	2.40	1.296	1.95	1.210
People would (or do) think I'm a wimp or not “macho” enough	2.24	1.219	1.43	0.937
I don't want people to stereotype me negatively (e.g., that I must be strange)	2.06	1.165	1.83	1.234
**Overall**	2.98		2.06	
**Unavailability and limited choice**				
Vegetarian options are not available where I grocery shop	2.34	1.343	1.81	1.051
There is too limited a choice when I eat out	3.40	1.341	2.47	1.258
I need more information about vegetarian diets	3.35	1.429	1.93	1.239
**Overall**	3.03		2.07	
**Unwilling or inability to change**				
Someone else decides most of the food I eat	2.40	1.383	1.46	0.965
It takes too long to prepare vegetarian food	2.07	2.070	1.93	1.174
I don't want to eat strange or unusual foods	2.45	1.309	1.43	0.891
I don't have enough willpower	3.01	1.393	1.98	1.144
I don't know what to eat instead of meat	2.41	1.224	1.48	0.975
I lack the cooking skills to change my diet that much	2.58	2.580	1.53	0.983
I don't want to change my eating habits or routine	2.80	1.347	1.76	1.225
**Overall**	2.53		1.65	

1, strongly disagree; 2, disagree; 3, neutral; 4, agree; 5, strongly agree.

Further, both urban and rural respondents agreed that traditional prejudice, peer pressure, unavailability of choices, and unwillingness or inability to change make it somewhat uncomfortable or difficult to adopt a vegetarian diet. Among data from urban ones, the highest mean score was for the barrier “My family eats meat” (2.70), followed by “There is not enough B12 in vegetarian diets” (2.68), and lastly “There is too limited a choice when I eat out” (2.47). In contrast, among rural respondents, the highest mean score was for the barrier “My family eats meat” (4.15), followed by “My friends eat meat” (4.06), and lastly “I like eating meat” (3.80). These data indicate that both types of respondents were greatly influenced by peers, especially friends and family members. Compared with urban tourists, their rural counterparts were mostly affected by friends and family members, which is typical of the deep-rooted family-based ideology and friendly neighborly culture prevalent in rural China (10).

To sum up, the responses to the multiple-choice questions on tourists' perceived barriers reflected that out of 206 respondents, 128 (62.1%) selected unavailability and limited choice as the most significant barrier. About 53.9% of the respondents considered peer pressure as the second barrier and 52.4% chose traditional prejudice and habit as the third highest barrier to vegetarianism. A further 44.2% mentioned unwillingness or inability to change, and other barriers including cooking skills and resource constraint were selected by just 1.9% of respondents.

#### Qualitative data comparison

In addition to the survey, open-ended questions were designed to collect participants' subjective information. At the end of each section of the questionnaire, respondents also had the opportunity to provide additional comments. Some of the comments received on other benefits of vegetarianism included factors such as having a clean conscience, being environment-friendly, living healthier, and having a better relationship with local food in general. Table 6 (below) summarizes participants' comments on the benefits of vegetarianism.

**Table 6:** Participants' comments on other benefits of vegetarianism.

**Theme 1: Personal health issue**
After consuming vegetarian, I am feeling lighter and stronger at the gym
It's amazing. I don't care about “trendy”. Since I am vegetarian my entire life changed.
I can eat more fiber, and change my unhealthy eating habit, this makes me more energetic
Vegetarians are less likely to have cardiovascular problems than meat-eaters
**Theme 2: Personal taste and choice**
I am living a more conscious lifestyle
I can live a little bit better in this world
Trying out new recipes all the time is awesome!
I learned so much more about nutrition than before.
It is very interesting to find new vegetarian replacement products! For example, for making a vegetarian ”cheesecake“, it's challenging but at the same time lots of fun.
I lost weight and becoming vegetarian makes me unbelievably happy
**Theme 3: environmental protection and resource conservation**
To me the most important thing about vegetarianism is that it helps with our environment issues.
Consuming vegetarian will reduce greenhouse gas
It will save the world and animals
Being a role model for my children. I do my job to save the planet for my children and grandchildren.
**Theme 4: Personal interest**
It got me into a better relationship with food in general
Milk causes acne for me
I avoid violence, by not eating animals who are abused in conventional farms, it can save the planet
It's better for the skin to eat dairy-free
Being a role model/positive influence on others
It helps to clear up my skin
Peace of mind
It is more aligned with my values
**Theme 5: Ethical consideration**
Just the thought of killing an animal for the pleasure of my taste feels so wrong.
It's not a trendy lifestyle; we do not need to eat animals to live.
I practice vegetarianism for global social and environmental justice.
I believe animal consumption harms animals harms our environment and harms people (health aspects) as well as the fact that work in meat and dairy “production” is largely done by marginalized and poor communities, affecting their mental and emotional wellbeing as well as air and water pollution.

Based on content analysis of participants' comments, two interesting findings emerged. First, expressions such as environmental-friendly, resource conservation, and animal protection are more frequently mentioned; more than 86% of respondents mentioned these factors. More than 30% of the respondents (n-62) confirmed that their vegetarian consumption was mainly for the public interest, rather than personal-related motivation. Second, in 101 (87%) out of 116 comments received from urban respondents, the main focus was ethical consideration, environmental protection, resource conservation, and climate change. This reflected that they were more concerned about external environmental protection and sustainable social development. However, on the other hand, only 62 (68.8%) out of 90 rural respondents focused on ethical consideration and environmental protection, and 10 (11.1%) respondents in rural areas mainly emphasize their nutrition structure improvement, fiber intake, losing weight, and long-term eating habits. This finding reflects rural tourists' emphasis on food and diet diversity.

Table 7 (below) summarizes the respondents' comments on other barriers to vegetarianism.

**Table 7:** Respondents' comments on other barriers to vegetarianism.

**Theme 1: Traditional prejudice and habit**
It must also be noted that vegetarianism is treated with prejudice in our culture completely - there are vegetarian traditions in many other countries and cultures but modern-day vegetarianism is largely seen as a privileged upper classes' movement which marginalizes people of remote villages.
Vegetables are contaminated with pesticides.
It is very expensive to buy organic vegetarian food
Eating vegetarian cannot meet nutritional requirement for human beings
The concept of balanced diet requires eating not only vegetables but also the meat
**Theme 2: Peer pressure**
I don't have vegetarian friend at all
I always have to justify myself while I am eating vegetarian
Some of my friends are annoyed of vegetarians
I have a meat-eating boyfriend
The public is ignorant of vegetarianism
**Theme 3: Unavailability and limited choice**
There are no vegetarian meals in my university
The vegetarian food in school cafeterias or normal supermarkets are often not clearly labeled – this means I often have to read through the ingredients, which is time-consuming and annoying
Limited access to vegetarian groceries
The fact that vegetarian foods are more expensive
There are no plenty of good vegetarian restaurants. The real vegetarian restaurants in my city can only be found at canteen of temples.
It is difficult to purchase the vegetarian ingredients in some rural cities
**Theme 4: Unwillingness or inability to change**
Lack of time to make my own food
It takes much longer for prepare the vegetarian diet, thus I am unwilling to accept
I still miss the taste of meet
It is difficult to keep being vegetarian

Traditional prejudice and habits, peer pressure, unavailability, and limited choice in a vegetarian diet were cited by 90% of the respondents as barriers. Of the 206 respondents, 185 (89.8%) mentioned unavailability and limited choice, 111 (53.8%) cited peer pressure, and 108 (52.4%) stated traditional prejudice and habit. However, one urban respondent remarked, “There is no barrier, only excuses.” This reflects how some urban Generation Z are actively supporting the vegetarian movement and are confident about this new consumption trend. More than 90% of the respondents insist on adopting a vegetarian diet their whole life after acceptance. In rural areas, however, more than half of the respondents are not so confident about the new trend, as one participant replied: “It seems extreme for many. It would be more attractive for many people to motivate them to reduce the consumption of animal products as a first step.” Rural participants are confused by the traditional concept of a balanced diet, and they have a long history of consuming animal products. Therefore, they seem to face greater barriers when choosing vegetarianism. Almost every rural respondent claimed that they may face greater challenges of vegetarian consumption during travel compared with urban ones.

Through content analysis, this study identified high-frequency words used to explain barriers to vegetarianism. This included limited plant-based options, unavailability, high search costs, the temptation of social circle, family members' request, and peer pressure. However, when asked about prospects, 90% of them remained positive about vegetarian consumption, and almost everyone advocated the promotion of vegetarian consumption during travel.

### Hypotheses testing

**Figure 1:**
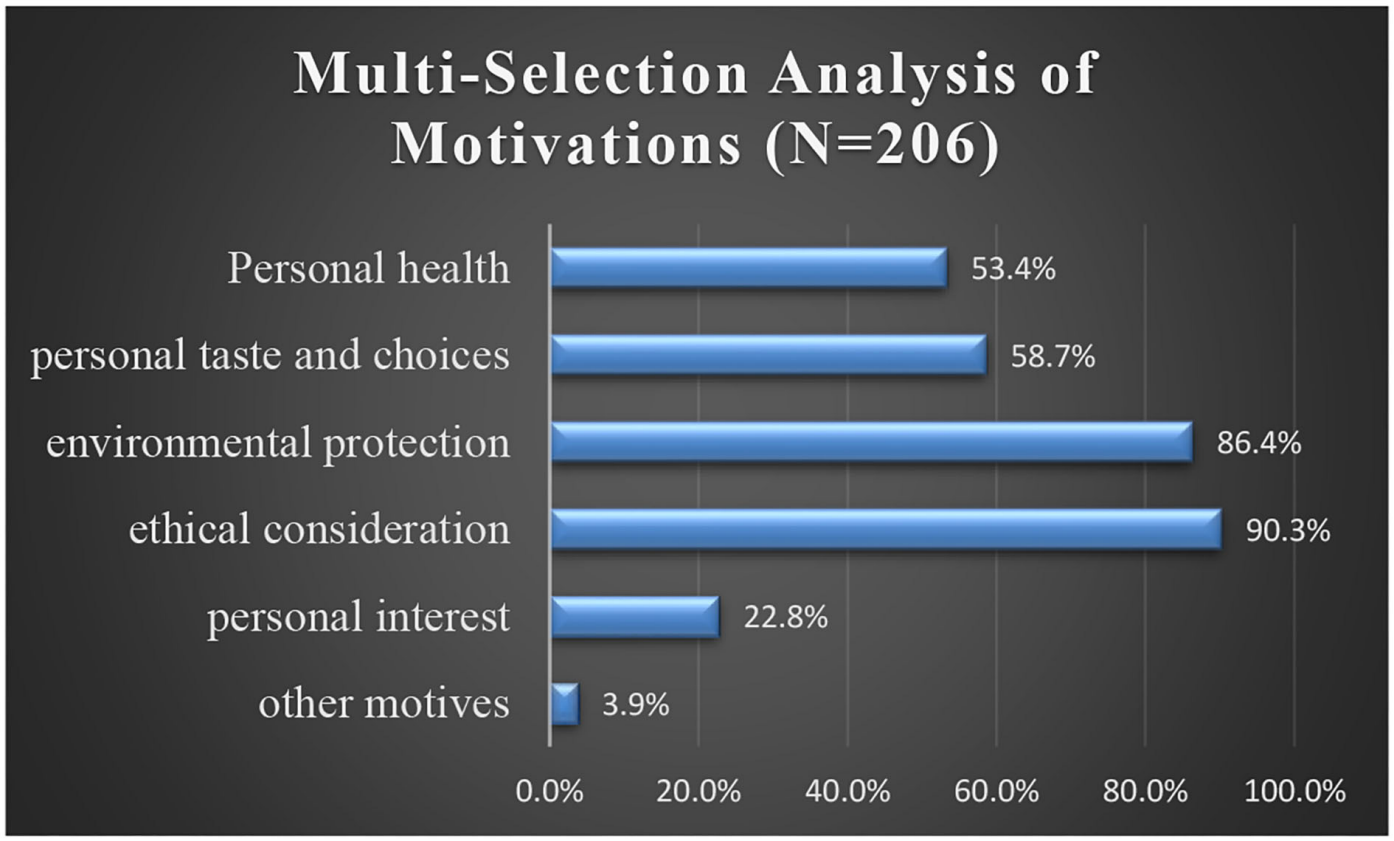
Motivations for adopting a vegetarian diet.

The perceived benefits and barriers were ranked based on mean utilizing descriptive statistics (see Table 8). The top four motivations were mainly related to environmental protection and resource conservation (4.19), ethical consideration (3.76), personal taste and choice (3.69), and personal health (3.52), and all these benefits contributed to vegetarian consumption positively. Second, the quantitative outcome of Figure 1 illustrates participants' most-frequently articulated motivations as ethical consideration (90.3%), personal taste, and environmental and resource motivation (86.4%). Thirdly, the qualitative analysis demonstrated that 86% of the participants were vegetarian mainly for the public interest, rather than personal-related motivation.

**Table 8:** Perceived benefits sorted by importance.

**Items**	**Score**
Environmental protection and resource conservation	4.19
Ethical consideration	3.76
Personal taste and choice	3.69
Personal healthcare issue	3.52
Personal interest	2.43

1, strongly disagree; 2, disagree; 3, neutral; 4, agree; 5, strongly agree.

Therefore, based on the above analysis, it can be stated that the external driving factors of environmental protection and ethical consideration are the top priorities for vegetarians. Thus, H_2_ can be accepted.

Similarly, as seen from Table 6, the score of “personal interest” is only 2.43, which is below the average score of 3.0 (it represented the neural). Taken together with the representation in Figure 1, the response rate of personal interest-related motivation is 22.8%, which is much less than the others. Furthermore, the qualitative analysis outcome also proved that personal-related motivation was not compelling. Thus, H_1_ cannot be accepted, as for most respondents, personal interest did not contribute to their vegetarianism.

**Figure 2:**
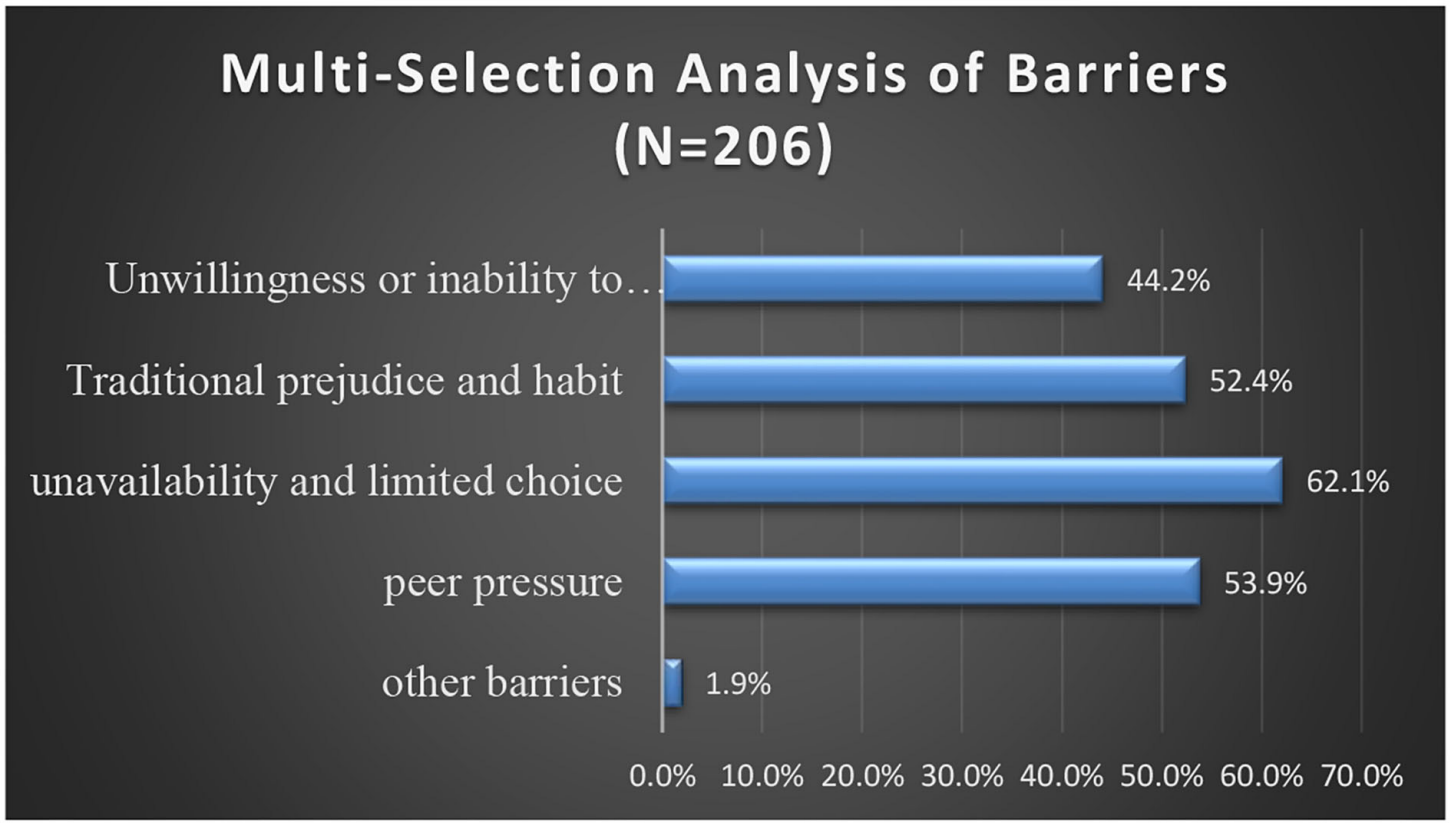
Barriers for adopting a vegetarian diet.

The top-ranking mean scores for perceived barriers to vegetarian consumption are presented in Table 9. The top three barriers were mainly related to unavailability and limited choice (2.49), peer pressure (2.46), and traditional prejudice and habit (2.35). This implied that the lack of availability of plant-based options and peer pressure had a stronger influence on vegetarian diet consumption. The quantitative outcome of Figure 2 also shows that the most frequently mentioned barriers by the respondents were unavailability and limited choice (62.1%), peer pressure (53.9%), and traditional prejudice and habit (52.4%). In addition, the qualitative analysis demonstrated that high-frequency words used by the respondents included limited plant-based options, unavailability, high search costs, the temptation of social circles, and traditional consuming habit.

**Table 9:** Perceived barriers sorted by importance.

**Items**	**Score**
Unavailability and limited choice	2.49
Peer pressure	2.46
Traditional prejudice and habit	2.35
Unwilling or inability to change	2.03

1, strongly disagree; 2, disagree; 3, neutral; 4, agree; 5, strongly agree.

From the above, it can be determined that the principal barriers were unavailability and limited choice, peer pressure, and traditional prejudice and habit, and they have a stronger negative influence on vegetarian diet consumption. Thus, H_5_ was accepted.

Although all these factors were said to pose a negative influence on the respondents' choice of vegetarianism, the scores for these factors were all below 3.0. This indicated that these factors were weak correlations with perceived barriers, and had a relatively limited weak influence on customers' vegetarian consumption. In addition, the qualitative outcome also revealed that 90% of the respondents were positive about the future of vegetarian consumption although some barriers existed. Thus, the negative influence of these barriers was relatively low for the respondents. Therefore, combining the quantitative and qualitative outcomes together, it can be concluded that H_4_ was not accepted.

The ranking of mean scores for perceived benefits and barriers to vegetarian consumption between rural and urban respondents is listed in Table 10. For the rural respondents, the top three benefits were environmental protection and resource conservation (3.67), personal taste and choice (3.44), and ethical consideration (3.36). This result was somewhat similar to urban respondents whose top three benefits were environmental protection and resource conservation (4.56), ethical consideration (4.05), and personal taste and choice (3.81). Two benefits including environmental protection and resource conservation and ethical consideration were included in both groups, which reflected that both have similar perceptions of motivations regarding vegetarian diets. The subtle difference was in that the average score of urban respondents was higher than their rural counterparts, which indicated that the motivation power in the urban areas was bigger. In addition, the results also demonstrated that urban vegetarians emphasized more on the benefits of environmental protection and ethical consideration, while rural vegetarians emphasized environmental protection and food and taste.

**Table 10:** Perceived benefits and barriers to vegetarianism between rural and urban respondents.

**Perceived benefits**		
	**Rural**	**Urban**
Personal health issue	3.23	3.73
Personal taste and choice	3.44	3.81
Environmental protection and resource conservation	3.67	4.56
Personal interest	2.63	2.04
Ethical consideration	3.36	4.05
**Perceived barriers**		
	**Rural**	**Urban**
Traditional prejudice and habit	3.06	1.81
Peer pressure	2.98	2.06
Unavailability and limited choice	3.03	2.07
Unwillingness or inability to change	2.53	1.65

1, strongly disagree; 2, disagree; 3, neutral; 4, agree; 5, strongly agree.

The results of ANOVA (used to test the differences between the two groups) are presented in Table 11. It shows that, though participants' score on benefits was significantly higher for urban tourists (M = 3.638, SD = 0.527) than rural tourists (M = 3.266, SD = 0.354, *p* < 0.001), we found no significant differences between the urban and rural perception of benefits (F < F _crit_, *p* = 0.44142). These results suggest that there are similar perceptions about the benefits of adopting vegetarian diets in both urban and rural China. Both ethics-related and environmental protection-related motives contributed positively and equally to their perceptions, and they have equal importance among rural and urban respondents. Thus, H_3_ was not accepted.

**Table 11:** One-Way ANOVA of perceived benefits between rural and urban respondents.

**Model summary**						
**Group**	**Count**	**Sum**	**Mean**	**Variance**		
Method 1	5	16.33	3.266	0.15203		
Method 2	5	18.19	3.638	0.90287		
**ANOVA**						
**Sources**	**Sum of squares**	**df**	**Mean square**	***F***	***P*** **-value**	**F crit**
Between Groups	0.34596	1	0.34596	0.6559105	0.44142	5.31766
Within Groups	4.2196	8	0.52745			
Total	4.56556	9				

Concerning the comparisons of perceived barriers, the top three barriers to vegetarianism among rural participants were traditional prejudice and habit (3.06), unavailability and limited choice (3.03), and peer pressure (2.98). The orders of these barriers were somewhat different from urban perceived barriers which were unavailability and limited choice (2.07), peer pressure (2.06), and traditional prejudice and habit (1.81). This indicated that although rural and urban respondents have similar perceived barriers, they differ in their levels of influence. While rural respondents view traditional prejudice and unavailability and limited choice as the top two barriers, urban respondents considered unavailability and limited choice and peer pressure as the top two barriers. In addition, the score of rural respondents was much higher than the urban scores, indicating that the former faced a higher level of barriers and challenges to vegetarianism.

The result of testing the differences in terms of perceived barriers using ANOVA is presented in Table 12. The results show that the participants' score on barriers was significantly higher for rural tourists (M = 2.900, SD = 0.314) than for urban tourists (M = 1.898, SD = 0.216, *p* < 0.001). Moreover, we observed significant differences between urban and rural perceptions of barriers (F > F crit, *p* = 0.00079). These results mean that rural participants experienced a higher intensity of barriers compared with urban participants concerning vegetarianism. Thus, H_6_ was accepted.

**Table 12:** One-way ANOVA of perceived barriers between rural and urban respondents.

**Group**	**Count**	**Sum**	**Mean**	**Variance**		
Method 1	4	11.60	2.900	0.06193		
Method 2	4	7.59	1.898	0.04169		
**ANOVA**						
**Sources**	**Sum of squares**	**df**	**Mean square**	***F***	***P*-value**	**F crit**
Between Groups	2.0100	1	2.01001	38.79397	0.00079	5.98737
Within Groups	0.3109	6	0.05181			
Total	2.3208	7				

1, strongly disagree; 2, disagree; 3, neutral; 4, agree; 5, strongly agree.

Rural vegetarians consider traditional prejudice and habit as the most important barrier, but for urban vegetarians, unavailability and limited choice were accorded priority. The two groups have different understandings and priority sequencing order and these results are also consistent with the conclusions above. The results from comparing the qualitative data indicate that both urban and rural respondents felt peer pressure, but urban respondents worried more about plant-based diet availability, whereas their rural counterparts paid much attention to traditional prejudice and historical eating habits. This was the reason behind the huge difference between them in terms of perceived barriers. Rural participants admitted that they might face greater barriers arising from traditional consuming habits, and the change needs great willpower. However, urban respondents had difficulties in searching for plant-based options. Combing the quantitive and qualitative results, the perceived barriers to vegetarianism to both groups were quite different and thus H_6_ was accepted.

The two groups had a different understanding and priority sequencing order which is evident from the quantitative data; the motivational scores of urban respondents were generally much higher than the rural respondents, while the scores for barriers were much higher among the rural respondents. In addition, based on the qualitative data comparison, the results indicated that both urban and rural respondents were motivated by environmental and ethical issues, though rural respondents may face greater challenges in pursuing their vegetarian passion. The open-ended interview results indicated that 90% of the respondents were likely to continue on a vegetarian diet their whole life after adopting vegetarianism. However, in rural areas, more than half of the respondents were not so confident about continuing this new trend. Compared with urban respondents, rural ones had to face greater barriers arising from long-term meat-eating habits as well as peer pressure. Thus, urban tourists had a higher sense of acceptance toward vegetarian consumption. Therefore, H_7_ was accepted.

Table 13 provides a summary of the hypotheses testing. In short, H_2_ and H_4_ are accepted, H_1_ and H_3_ are partially accepted, and H_5_ is rejected.

**Table 13:** Summary of hypotheses testing.

**Hypotheses**	**Result**
H_1_: Personal health, personal taste and choices, environmental protection and resource conservation, personal interests, and ethical consideration are positively related with Generation Z tourists' willingness to follow a vegetarian diet.	Partially accepted
H_2_: In general, of all the benefits, ethical-related and environmental protection-related motives have stronger positive effect on vegetarian consumption willingness.	Accepted
H_3_: The positive influence of Ethical-related and environmental protection-related motives are stronger for tourists in urban areas than those in rural ones.	Objected
H_4_: Traditional prejudice and habit, peer pressure, unavailability and limited choice and unwillingness or inability to change are negatively related with Generation Z tourists' vegetarian consumption	Partially accepted
H_5_: In general, among all the barriers, unavailability and limited choice, and peer pressure as well as traditional habit and prejudice have stronger negative influence on vegetarian diet consumption.	Accepted
H_6_: The negative influence of traditional prejudice and habit and unavailability and limited choice are higher for tourists in rural areas than those in urban ones.	Accepted
H_7_: Urban tourists (vs. rural ones) results in higher sense of acceptance toward vegetarian consumption.	Accepted

## Discussion, conclusion, and recommendations

### Discussions

#### Perceived benefits

This research showed that the Generation Z tourists' perceived benefits related to vegetarian consumption included improving animal welfare, increasing food taste and choice, protecting the environment, and improving personal health. Personal interests proved to be not positively related to participants' perceived benefits. Environmental protection, animal welfare improvement, and personal demand for multiple food choices were considered the most important benefits. Health-related benefits as well as personal interests ranked relatively low.

This result was just somewhat different from the findings of several studies (13, 15, 21) that were conducted in western countries. In their study, personal health benefits topped as the primary motivation, rather than environmental protection or moral considerations. As to the reasons behind it, firstly, as an oriental and traditional country favoring collectivism (23, 41), the Chinese people are wired to place collective concern as their first priority. Environmental consciousness among the public became a widespread concern since the proposition of “Lucid waters and lush mountains are invaluable assets” in 2005. Therefore, as post-2005 college students, it is understandable that they place environmental protection and ethical consideration in the first place while taking a trip. Second, as Glick-Bauer and Yeh stated, the concept of animal rights protection spread from 2013 onwards, and this particular demography pays much importance to the concept and has been influenced by it deeply (42).

The findings of this study are also similar to the findings of several other studies (14, 16–18) most of which stated that ethical factors were the top motivational factors. With increasing incidents of animal cruelty being reported on the internet and social media, people were getting more and more concerned about animal protection and sustainable development in society (43–45). Thus ethical benefits have also emerged as a top priority (46–48) for people embracing vegetarianism. The results of this research are also similar to the finding of Jansen et al. (19) who reported that people placed importance on ethical consideration and environmental protection first and lastly on personal health.

#### Perceived barriers

The main perceived barriers to adopting a vegetarian diet, ranked in the order of importance as expressed by the respondents are: unavailability and limited choice (2.49), peer pressure (2.46), traditional prejudice and habit (2.35), and lastly, unwillingness or inability to change (2.03). The lack of information about plant-based diets and limited choices was viewed as the strongest barrier. Vegetarians often face a dilemma of relatively few plant-based diet options when compared with others who eat meat. In fact, because of its small market share in the food industry, the number of vegetarian restaurants is relatively less. Together with high search costs and inconvenience, unavailability becomes the main concern for vegetarians. This finding is consistent with previous research (17, 26, 28, 29) that found that while eating out, the choices for vegetarian food were too limited and lacked the availability of plant-based options.

The influence of peers is also consistent with the findings of recent studies (27, 30) which indicate that family/spouse/partner's meat-eating habits inhibit an individual's vegetarian consumption most. This research supplements Radnitz's research that found family members, friends, and partners influencing vegetarian consumption and their importance of influence were ranked as family members first, friends second, and partners ranked third (17, 49).

Traditional prejudice and habit were also proven to have a negative influence on people's vegetarian choices. Three studies conducted in western countries including the US, Australia, and South Africa had similar results. In Lea and Worsley's study, the main barrier to vegetarianism was satisfaction with meat eating and not being able to give it up (25). In Wieliczki's study, unwillingness or inability to alter their current dietary patterns was among the top three barriers to vegetarian consumption (27). This finding is consistent with the work of Radnitz et al., which stated that “perception that humans are ‘meant' to eat meat' and “I think humans are meant to eat meat” among to barriers to vegetarianism. Therefore, people's enjoyment of eating meat and their unwillingness to change their daily diet is also considered the main barriers (17).

The results of this current research are proven to be consistent with previous research conducted by Lea and Worsley (25), Wieliczki (27), and Radnitz et al. (17), especially the finding that although the vegetarian movement has been ongoing for a long time, significant barriers hindering people to adopt vegetarianism still exist and cannot be eliminated completely (50–52).

#### Perceived benefits and barriers between rural and urban respondents

As to the perceived motivation for vegetarianism among rural and urban post-millennials, this research found that both groups were motivated by environmental and ethical consciousness which played an equally important role in their decision to become vegetarian. However, subtle differences between the two groups exist; compared with rural vegetarians, urban ones had a better positive understanding of being vegetarian. The reasons may be rooted in the fact that vegetarian consumption started earlier and spread wider in urban regions than in rural areas. This result is consistent with the research by Liu, which noted that urban areas have become the leading force in the ' vegetarian revolution', and it also found that ”since 2016, most urban young adults, in general, became increasingly aware of the benefits of plant-based nutrition in China“ (53).

As to their perceived barriers, this research concluded that traditional prejudice, peer pressure, resource unavailability, and unwillingness or inability to change, all pose negative influences on young people's decision to adopt a vegetarian diet. The biggest difference is that urban vegetarians consider unavailability and limited choice as their foremost barrier, while rural ones view traditional prejudice and habit as their top barrier. This finding, however, is not in line with the research by Memon et al. (32) and He et al. (36). Both quantitative and qualitative results demonstrated rural participants perceive facing greater barriers from traditional consuming habits as well as limited choices, and the change needs great willpower. Under the pressure of long-term meat-consumption habits, together with limited vegetarian diet options, rural vegetarians find it more difficult to follow a vegetarian diet during their travels. Furthermore, compared with urban participants, the rural vegetarians gave a higher appraisal score of perceived barriers to adopting a vegetarian diet. It can be concluded that rural consumers face much more barriers while adopting a vegetarian diet, and this finding may enrich regional comparison literature on vegetarian consumption.

### Conclusions

This study aimed to explore Generation Z tourists' perception of vegetarianism, including their perception of benefits and barriers. A self-administrated questionnaire was administered for collecting feedback from 206 participants. Of them, 110 participants were from urban China, and 96 participants were from rural China. All of them were vegetarians aged 18 to 27. Data analysis revealed that people choose a vegetarian diet mainly for environmental protection, animal welfare, food and taste, and personal health. Data comparison found that both rural and urban vegetarians put more emphasis on the benefits of environmental protection and ethical consideration, and the subtle difference between them is that rural vegetarians also emphasized food and taste. The reason is rooted in the fact that urban societies have a longer history of vegetarianism and young people are getting more and more concerned about environmental and ethical issues. The participants' perceived barriers, listed in the order of their priority are unavailability and limited choice, peer pressure, traditional prejudice, and unwillingness or inability to change. The former two were viewed as the strongest barriers to vegetarianism in general. The difference between them is that urban vegetarians consider unavailability and limited choice as their top-most barrier, while rural vegetarians view traditional prejudice as the main barrier. Due to the pressure of traditional dietary habits and peer influence, rural tourists face much more challenges when adopting a vegetarian diet. And compared with urban tourists, rural ones have a lower sense of acceptance toward vegetarian consumption.

### Theoretical contributions

This research contributes to the literature in three aspects. First, although previous studies have discussed consumers' perceptions of motivation and barriers to vegetarianism consumption, most of them were conducted in western countries. Our research investigated these factors in the Chinese context, adding to the relatively few studies in the literature. Our study extends the understanding of vegetarianism in China. Secondly, recent research has investigated micro-level aspects of benefits and barriers to vegetarian consumption in different regions, and our study goes further to make a comparison and contrast analysis of vegetarians from urban and rural areas. Thirdly, in addition to investigating their motivation and barriers, our work also goes further to establish that rural vegetarians face greater challenges during their travel.

### Practical implications

Vegetarian consumption during travel has become a primary concern for vegetarian tourists and deserves additional efforts from both tourist operators and the tourism administration department. As to the business practitioners, the categories of vegetarian diet should be increased and a variety of options should be put forward for vegetarian tourists, besides green production, green packaging, as well as green marketing, should also be adopted for meeting these tourists' demand for environmental protection and resource conservation. Third, reasonable planning schemes should be planned to ensure enough availability of vegetarian providers.

Urban regions have long been viewed as the leading force of vegetarianism in China, and rural regions, with a large population of postmillennial consumers, have gradually realized and accepted the importance of plant-based diets.

As for the tourism administration department, additional efforts should be made to alleviate the most commonly perceived barriers to vegetarianism, i.e., vegetarian counseling and education (53), vegetarian exhibitions, vegetarian lectures, and so on (54–56). Public health departments should also adopt some measures to provide enough information about vegetarian diets and address the public's concerns about them (54, 57). Gradually, the benefits of vegetarian consumption will be understood by generation Z consumers both in urban and rural areas. This would ensure that a nutrition-balanced diet has a bright future in China.

### Future research

Few researchers have investigated micro-level aspects of benefits and barriers to vegetarian consumption in different regions, and this present study focuses on perception differences in vegetarian consumption among Generation Z tourists. While on the other hand, this research also has some limitations and needs to be improved in the following aspects: (a) data should be collected from a larger sample of the population. It is suggested to examine their perceived differences in a sample of a broader age range, not just limited to millennials. In fact, due to work pressure and worrisome health conditions, more and more middle-aged populations are turning to veganism. Thus, to have a detailed investigation of their motivations for a change in their diet is also necessary. (b) Future research should also examine the relationship between consumers' perception and their demographic factors such as work status, living conditions, and income, which may have a greater influence on people's perception of vegetarianism. Thus, it is necessary to conduct studies to enrich the theoretical and empirical research on this subject in the future. (c) cross-culture and cross-country perceptional differences in vegetarian consumption is a subject worthy of study. Implications can be drawn for developing effective interventions for healthy and pro-environment dietary patterns.

## Data availability statement

The original contributions presented in the study are included in the article/supplementary material, further inquiries can be directed to the corresponding author.

## Ethics statement

Ethical review and approval was not required for the study on human participants in accordance with the local legislation and institutional requirements. Written informed consent from the participants was not required to participate in this study in accordance with the national legislation and the institutional requirements.

## Author contributions

Conceptualization and validation: GC and NR. Methodology and project administration: GC. Software, investigation, and visualization: WT. Formal analysis, resources, and supervision: BY. Data curation: NR. Writing—original draft preparation: GC. Writing—review and editing and funding acquisition: JZ. All authors have read and agreed to the published version of the manuscript.
